# Light People: Prof. Eric Mazur speaks about ultrafast optics and education

**DOI:** 10.1038/s41377-024-01402-8

**Published:** 2024-02-26

**Authors:** Chenzi Guo, Yang Li

**Affiliations:** 1 Changchun Institute of Optics, Fine Mechanics and Physics, Chinese Academy of Sciences, Changchun, China; 2 State Key Laboratory of Precision Measurement Technology and Instrument, Department of Precision Instrument, Tsinghua University, Beijing, China

**Keywords:** Nonlinear optics, Metamaterials

## Abstract

Prof. Eric Mazur is a great influencer over and beyond the optics community. As a physicist, he is a pioneer of ultrafast optics and was one of the inventors of colliding-pulse mode-locked laser. As an educator, he not only gave talks to thousands, but also revolutionized teaching with his globally renowned methodology “Peer Instruction”. As a leader and entrepreneur, he co-founded several companies and was President of Optica (formerly the Optical Society) and currently is the Chair of the Optica Foundation. Here, *Light: Science & Applications* talked with Prof. Eric Mazur about his opinions on research, education and industry. The full interview video can be found in the Supplementary File.

**Figure:**
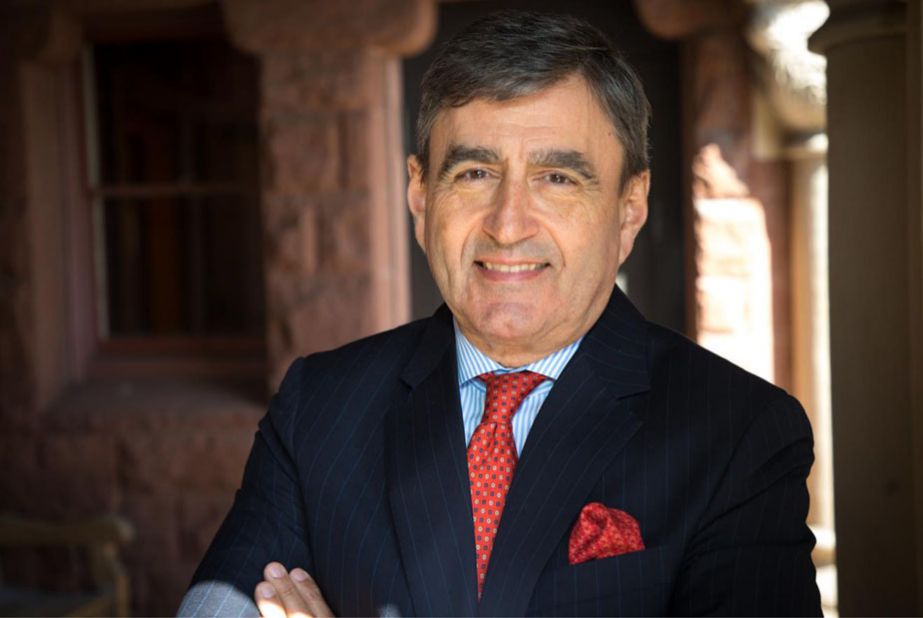
Eric Mazur

**Short Bio of Prof. Eric Mazur**

Prof. Eric Mazur is the Balkanski Professor of Physics and Applied Physics and Academic Dean of Applied Sciences and Engineering at the John A. Paulson School of Engineering and Applied Sciences at Harvard University, Member of the Faculty of Education at the Harvard Graduate School of Education, and Past President of Optica (formerly the Optical Society). He is a Member of the Royal Academy of Sciences of the Netherlands and a Member of the Royal Holland Society of Sciences and Humanities. He is a Fellow of AAAS, Optica, APS, and the American Association of Physics Teachers.

Dr. Mazur has made significant contributions to spectroscopy, light scattering, the interaction of ultrashort laser pulses with materials, and nanophotonics. Dr. Mazur has served on numerous committees and councils, including advisory and visiting committees for the National Science Foundation, and has chaired and organized many national and international scientific conferences. He serves as a consultant to industry in the electronics and telecommunications industry, and has founded many companies, including SiOnyx, Learning Catalytics, which was acquired by Pearson, and Perusall.

In addition to his work in optical physics, Mazur has been very active in education. In 1990 he began developing Peer Instruction, a method for teaching large classes interactively. He is the author of Peer Instruction: A User’s Manual (Prentice Hall, 1997), a book that explains how to teach large lecture classes interactively. In 2006 he helped produce the award-winning DVD Interactive Teaching. Dr. Mazur’s teaching method has developed a large following, both nationally and internationally, and has been adopted across many disciplines.

Dr. Mazur has received numerous awards, including the Esther Hoffman Beller award from Optica and the Millikan Medal from the American Association of Physics Teachers. In 2014 Mazur became the inaugural recipient of the Minerva Prize for Advancements in Higher Education. He is the author or co-author of 380 scientific publications, 52 patents, and several books.

**Q1: Your group pioneered in femtosecond laser nano- and microfabrication, could you share how you started?**

A1: Many of the most interesting developments in my career occurred more or less by accident. This is one of them: about 20 years ago we started focusing ultrashort laser pulses in transparent materials, and we discovered we could create a plasma inside the bulk of the material, which was surprising and interesting then. The reason we obtained a plasma was because a microscope objective focused light very tightly, and it turned out the focusing was really important for the generation of a plasma. From that moment on, many groups started to do femtosecond laser writing. My group did a number of foundational experiments on femtosecond micromachining of integrated optical devices. Later, drifted out of inorganic, solid materials (like glass) and towards more biologically relevant materials, using lasers to micro-manipulate the machinery of life. Currently, we are no longer active in this field — the field of femtosecond laser micromachining has grown enormously. I like to start new things, but once many people are doing it, I get out of it.

**Q2: You published a review paper on zero-index materials in** ***Light: Science & Applications*, and I really enjoy reading that. What do you think are the potential applications for zero-index materials?**

A2-Eric: Maybe I’ll list a few. In any physical expression, where the index appears in the numerator or the denominator, you can expect unusual phenomena. One such area is nonlinear optics. In fact, in collaboration with Boyd’s Group, we showed relaxed phase matching in a zero-index material. Another area of interest is in quantum optics. One of my graduate students just demonstrated that zero-index materials give rise to superradiance in a diamond-based zero-index metamaterial. Imagine you have a block of zero-index material, and you have two embedded emitters that emit exactly at that zero-index frequency. Spontaneous emission is completely suppressed, stimulated emissions enhanced. In a sense, these two emitters are completely entangled, and can only emit at the same time, giving rise to superradiance. Yang may want to add some additional thoughts.

A2-Yang: To expand on what Eric said, the collaboration between Robert Boyd and Eric’s group facilitated the phase-matching condition of four-wave mixing in a silicon-based zero index structure. But the issue is that the phase matching in four-wave mixing always happens at 4 different, but very close wavelengths. Therefore we cannot typically generate a nonlinear frequency component that has a very different frequency from the pump frequency. So, our group is now working on spontaneous four-wave mixing in zero-index materials. Based on a similar principle, we reduced the pump intensity to a weak pump regime, and then we were able to produce entangled photon pairs or single photons. Subsequently, we demonstrated a zero-index metamaterial as entangled photon pair source with higher nonlinear conversion efficiency. To achieve this, we need phase matching and a high nonlinear index which is proportional to the impedance. For zero-index materials, matched impedance is higher than that of a silicon waveguide. So we can achieve better nonlinear conversion efficiency.

**Q3: Many researchers teach, but being an educator who can build and lead research on education is something different. How did you make this transition from educator to education researcher?**

A3: Well. I simply realized that one can be as scientific about teaching as one is in the lab. In the lab, we practice science, write hypotheses, collect data, and analyze the data. We either refuse the hypothesis or accept it, and then continue to advance our knowledge of how nature works. But the same can be done in the classroom. So back in 1990s, I started to collect and analyze data in my classroom, and I realized that some of my students, in spite of being able to pass exams, were unable to answer very simple questions about Newtonian mechanics. And I asked myself, how is that possible, and what can I do about it? So, since that moment, for the past 32 years, I have actually used my classroom as an extension of my lab. In the lab, I work with photons and lasers. In the classroom, I work with knowledge and students. And I think that there should not really be a separation between these two. And you know, we really have a moral obligation to educate. Doing research without transferring that knowledge to the next generation of scientists or explaining the importance of science to non-scientists would be a big mistake. Because ultimately, it’s society and not the scientific community which determines the future of science. We need to be supported by an educated society.

**Q4: As a highly prestigious educator, much of your knowledge and skills must have been very intuitive to you. How do you manage to summarize all the confusion and simple questions that your students don’t understand? For some first learners, sometimes it’s even difficult for them to verbalize their questions?**

**Figure:**
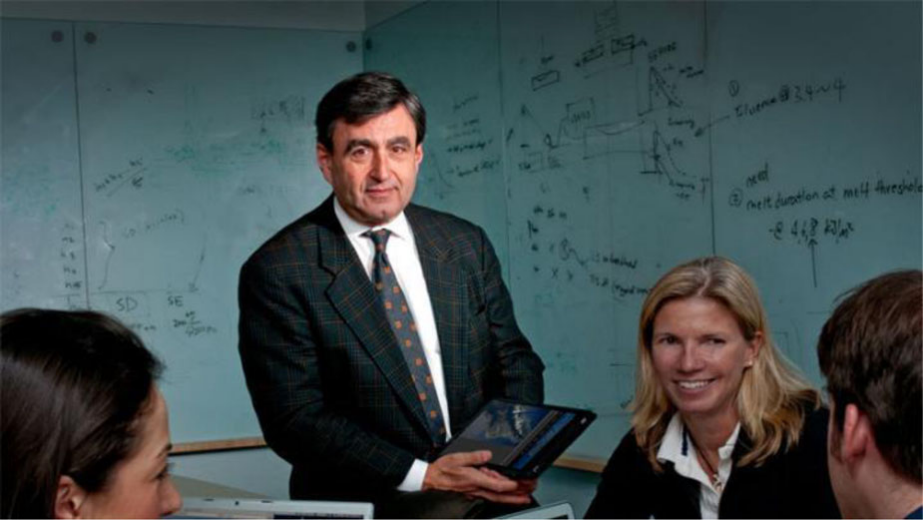
Eric Mazur in education research

A4: Absolutely! Good question. If you don’t understand something, it’s very hard to articulate what you don’t understand. The other problem is that when you become an expert at something, you no longer realize that others don’t know many things that are obvious to you. You have long forgotten how you learned it yourself. So, the first step in improving education is to find out what the students don’t understand. And I stumbled on that by accident. I started to ask really simple questions. Let me give you an example. If I ask the student, what is Newton’s third law? They all say “the force exerted by object 1 on object 2 is equal to the force exerted by object 2 on object 1. And if I give them a problem where they have to use Newton’s third law equation, there’s no problem either. But if I ask a daily life question, such as “A heavy truck collides with a light car on the highway. Is the force exerted by the car on the truck larger than that of the truck on the car?” then all of a sudden you find that almost all students believe that heavier objects exert a larger force on lighter ones than the other way around. That’s because they have not completely internalized the meaning of the concept of force. I noticed that my students could apply Newton’s Law in a textbook problem, but they could not recognize it in the world in which we live. So that led me to think “how can I solve that”? And I realized I was not the best person to help them, because I learned it such long time ago and I can almost intuitively answer daily life questions. I have forgotten how a beginning learner thinks. That gave me the idea of students helping each other improving their understanding, with stronger students helping weaker students master concepts.

**Q5: With your transformative teaching methodology, you published a book “Peer Instruction”, which transformed the way of educating and teaching. How did you come up with this genius idea? How did peer instruction evolve in the past few decades?**

**Figure:**
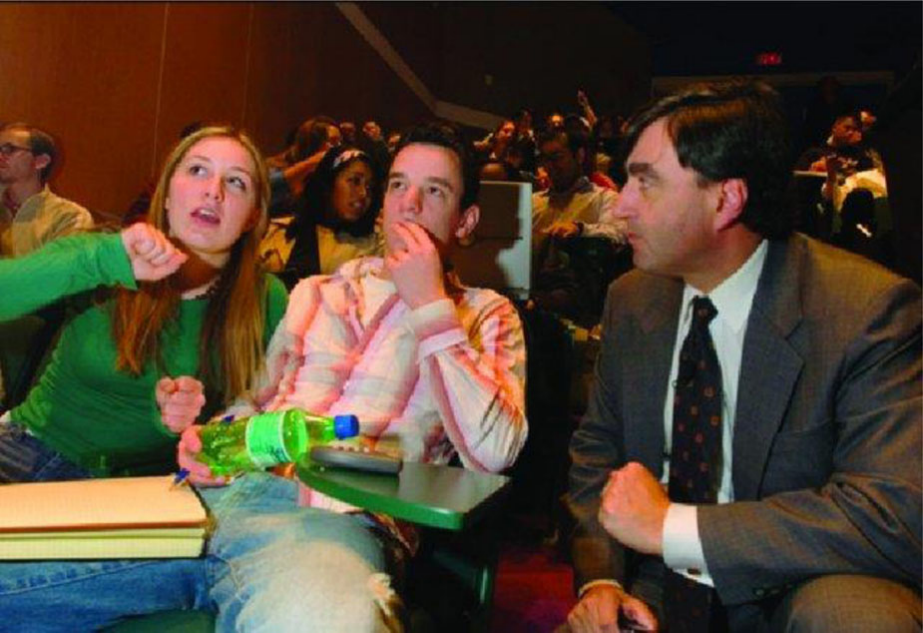
Eric Mazur in peer instruction

A5: It was serendipity, just like femtosecond laser micromachining. I told you about the question of the truck and light car colliding. And I remember me being shocked by the students’ wrong answers. So, I tried to explain the answer to the question using free-body diagrams and covering the board with equations. And then I simply said “By Newton’s third Law, these two forces are the same.” To me that was obvious, but I could see from their faces that they were mystified. So, I asked them, what are you confused about? But they couldn’t verbalize their confusion. At that point, I didn’t know what to do, but I knew some of the students had given the right answer. So I told the students, why don’t you just discuss it with each other? At that time I had a huge class of 250 students and they all started talking to each other. They forgot about me in front of the class. But what was really surprising to me was that within 3 min they figured it out, while I had taken tens of minutes without success. First, I thought, how is that possible? But then I realized that students who know the answer still are aware of the conceptual difficulties a beginning student faces because they only recently learned it, and therefore they are better positioned to help other students than Prof. Mazur in front of the class, who has long forgotten these difficulties. That’s essentially the basic idea of Peer Instruction. From that moment on, I started to teach by questioning, rather than by telling. I ask a question, ask them to think about it, commit to an answer, and then look for another student who has a different answer and try to convince that student that they are wrong. And the methodology very quickly took off. I demonstrated that using this approach I could double or even triple the learning gains in my classes. Once people discovered what I was doing in class, they asked me to give lectures about it, and eventually I wrote a book about Peer Instruction and at that point it sort of snowballed. One of the key features of Peer Instruction is active learning—learning doesn’t happen passively. You don’t learn to play the piano by watching somebody play the piano. You have to play the piano. Actually, I think you don’t learn anything by just watching or listening. I think that what Peer Instruction did was that it made it possible for many instructors around the world to switch from a passive to an active learning approach. Nowadays, there are many ways to bring active learning into the classroom, including team-based learning, problem-based learning, and project-based learning. Peer Instruction is just one form of active learning. But I think the reason that Peer Instruction was so important in transforming education was that it is so easily to implement. You can implement it in a lecture hall and you don’t have to change the learning space. I think that was probably one of the keys to the success of Peer Instruction.

**Q6: What is the largest audience you have ever addressed? And for how large an audience can you make Peer Instruction work?**

**Figure:**
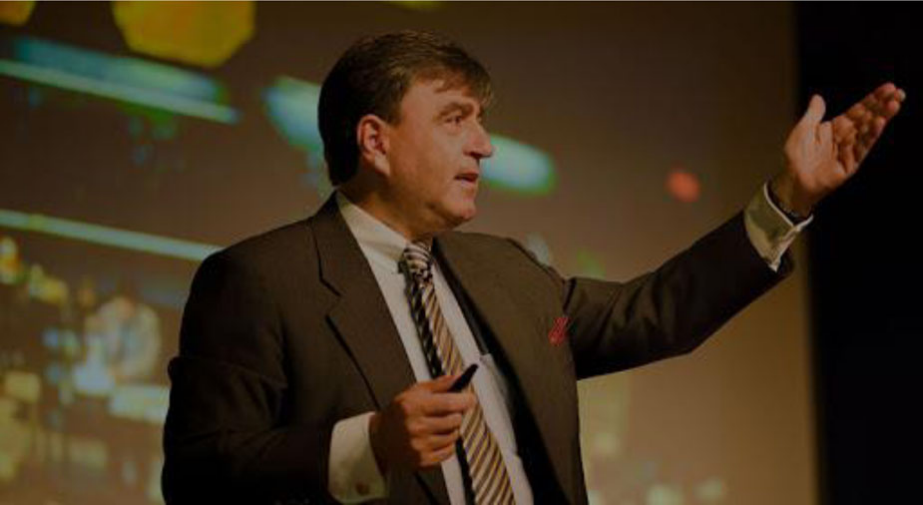
Eric Mazur gives a presentation

A6: A number of years ago I was at a conference in Brisbane, where I had an audience of 8,000 and I tried out Peer Instruction during my talk. It was amazing to see so many people become so enthusiastic about learning. But I wouldn’t recommend growing our classes to thousands of students. My classes typically have between 100 and 250 students, because the more students you have, the less you connect to them. The smaller the class, the better you get to know your students as a person. And I think connecting to your student as a person is very important, which is difficult in large classes. However, there will always be a need for large classes, in particular in introductory classes which tend to be large because they bring together many students from different backgrounds. That is where Peer Instruction is a great way of actively engaging a passive audience in a theater-like setting.

**Q7: Apart from teaching and researching, you have co-founded several companies, such as SiOnyx and Learning Catalytics with huge success, and you have also provided tremendous service to our optics community, including serving as the president of Optica. How did you manage to do all of this? And what, if any, suggestions do you have for the younger generation? And you are also an extremely passionate person. Where do you get the energy?**

A7: You are correct about me being passionate, and I believe that being passionate about something is very important. Another key aspect to success is that we should learn how to work effectively as a team. Working in science is very competitive, but even if you’re competitive as an individual, you can never accomplish more than those who work as a team. So, in all of my efforts, including my startup companies, it was important to put together a good team where I could help inspire people and provide a vision. But none of these visions could have taken off, if others had not contributed to it. Another motivator for me is service to the community. As you know, I was the President of Optica, and am now the Chair of the Optica Foundation. An important part of my life is to be of service to our optics community. We need to support the next generation and young professionals in optics by recognizing their accomplishments and making it possible for more people to have the opportunities that many of us have had. Finally, instead of micromanaging, I think it’s important to give people freedom and autonomy. As an individual, if we don’t have a feeling of autonomy and growth, it’s very hard to be productive. So autonomy is important for the success of the entire group or team.

**Q8: Do you have any suggestions for early career faculty members? Some put emphasis on research, and others are more oriented toward teaching and educating. What suggestions do you have for these two separate tracks?**

A8: Above all I think it’s important to remain curious, regardless of any track. We should go for scientific advances because of this innate curiosity of the human mind, rather than pursuing a field that looks hot right now. If you focus on what you are interested in, what makes you curious, follow your passion, and use that passion to inform your research or teaching, then that is going to advance your own career, science, and teaching the most. When I came to Harvard, I had a great job offer in industry, and my intention was to stay at Harvard as a postdoc for one or two years. I never intended to stay long. But now I have been at Harvard for 42 years in total. Because I thought I was going to go back to the Netherlands and accept my job in industry, I never tried to arrange my work to please others to obtain tenure at Harvard. I did what I thought was best for me. Now, I understand the pressure, the quest for tenure poses on many of my young colleagues, but my advice would still be, follow your passion — that is going to make you the happiest, and is also most likely to make you succeed.

**Q9: How do you mentor your PhD students? How do you balance happiness and pressure, and balance short- and long-term happiness?**

A9: I recently read about something called self-determination theory, which says that human beings have—in addition to food, air and other biological needs—three fundamental psychological needs. These are, first, the need to feel part of a community—a team, an organization, a club, or anything. Second, the need for a feeling of growth, of advancing and not staying in place. And lastly, a need of autonomy—that your growth takes place because of your own actions. These three psychological needs, community, growth, and autonomy, are fundamental to the human mind. While I certainly didn’t realize that back in the early days of my group, but when I think about what I did in my group, I certainly always worked on building a community, because that’s what my advisor did when I was a PhD student. The community is not just about working together, but also socializing and having fun together. I have also always given my PhD students much autonomy, supporting them in their growth rather than dictating what they need to do. I have always taken the mentoring of my PhD students very seriously and I take pride in the success they have accomplished after leaving my group.

**Q10: Your father, Prof. Peter Mazur, was one of the most prominent physicists at his time, and a cofounder of the non-equilibrium thermodynamics field. Was he the reason that you pursued a PhD degree and stayed in science?**

A10: My father definitely always stimulated my scientific thinking and understanding. However, the one who encouraged my curiosity and interest in science and mechanical things during my childhood was my grandfather, a civil engineer. He would come to visit our home with a box of transistors, resistors, and metal toy construction sets. At age 10, I started tinkering with electronics. I built a radio, a synthesizer, etc. My father was a theorist. He was a great scientist, but he was not an experimentalist of any kind. When I went to university, my father always gave me great advice, he advised what group I should join for my PhD. I’m eternally grateful to him because the group he recommended was a fantastic group where I learned all the skills that were essential to build my own group later. After I finished my PhD, I really wanted to go and work in industry, and I lined up a job at Philips in Eindhoven, which at that time, was similar to Bell labs. But my father convinced me to take a short-time postdoc in the US first. As I had never been to the US, I thought that might be an interesting thing to do. When I asked where should I apply, he suggested a great list, all of which were Nobel Laureates! I applied, and I got a job offer from Prof. Nicolaas Bloembergen (1981 Nobel Prize Winner in Physics) at Harvard, and that completely changed my life plans, as I still am at Harvard. So, my father had a major influence on me, but I would also give credit to my grandfather for stimulating the experimental aspects of my interest.

### Supplementary Information

Supplementary video

